# Gestational prognostic factors in women with recurrent spontaneous abortion

**DOI:** 10.1590/S1516-31802006000400002

**Published:** 2006-05-04

**Authors:** Marcos Roberto Caetano, Egle Couto, Renato Passini, Renata Zaccaria Simoni, Ricardo Barini

**Keywords:** Habitual abortion, Allergy and immunology, Autoimmunity, High-risk pregnancy, Immunotherapy, Aborto habitual, Alergia e imunologia, Auto-imunidade, Gravidez de alto risco, Imunoterapia

## Abstract

**CONTEXT AND OBJECTIVE:**

Recurrent spontaneous abortion (RSA) is defined as three or more consecutive pregnancy losses before 20 weeks and is associated with several etiological factors related to genetics, anatomy, hormones, infections and immunology, for example. Many cases of RSA remain unclear. New factors or their associations may influence gestational results. The aim was to identify possible single or associated causes of RSA that could predict gestational prognosis for women undergoing investigation and treatment.

**DESIGN AND SETTING:**

Case-control study, at the Recurrent Abortion Outpatient Clinic, Department of Obstetrics and Gynecology School of Medicine, Universidade Estadual de Campinas (Unicamp).

**METHODS:**

Two hundred and forty-six medical records of women with RSA seen at the Recurrent Abortion Outpatient Clinic, Department of Obstetrics and Gynecology School of Medicine, Universidade Estadual de Campinas (Unicamp), between 1994 and 2003, were evaluated. Data on age, obstetric history, possible etiological factors, treatment and pregnancy outcomes were evaluated. Statistical analysis was performed using odds ratios (OR), logistic regression analysis and decision trees.

**RESULTS:**

Two hundred and twenty-nine women were included in the study. The most frequently found etiological factors were immunological, particularly alloimmune factors (93.9%). Women with a single alloimmune factor had better gestational results (77.7% deliveries) than those with other associated factors. Autoimmune factors were associated with a higher abortion rate (OR: 4.30; 95% confidence interval, CI: 1.36-13.63). No association was found between the number of abortions prior to treatment and pregnancy results. Women aged 40 or over presented the highest rate of spontaneous abortion (OR: 5.83; 95% CI: 1.12-30.40).

**CONCLUSION:**

Age over 40 years old, immunological factors and two or more concomitant factors were associated with poor gestational outcomes among the women studied.

## RESUMO

**CONTEXTO E OBJETIVO:**

A perda espontânea de três ou mais gestações subseqüentes é chamada de aborto espontâneo recorrente (AER). É relacionado com alterações genéticas, anatômicas, hormonais, infecciosas, imunológicas e outras. Muitos casos de AER continuam como de causa desconhecida. Novos fatores ou associações podem influenciar o resultado gestacional. O objetivo do estudo foi identificar as possíveis causas do AER, isoladas ou associadas, que poderiam predizer o prognóstico gestacional em mulheres submetidas a um protocolo de investigação e tratamento.

**TIPO DE ESTUDO E LOCAL:**

Estudo de caso-controle, no ambulatório de Perdas Gestacionais do Centro de Atenção Integral a Saúde da Mulher da Universidade Estadual de Campinas.

**MÉTODOS:**

Foram revisados 246 prontuários médicos de mulheres com três ou mais perdas espontâneas sucessivas atendidas no Ambulatório de Perdas Gestacionais do CAISM/Unicamp entre 1994 e 2003. Foram avaliados dados relativos à idade, antecedentes obstétricos, possíveis etiologias para a recorrência do aborto, tratamentos realizados e resultados gestacionais. A análise estatística envolveu razão de chances (RC), análise por regressão logística e arvores de decisão.

**RESULTADOS:**

229 mulheres foram incluídas no estudo. O fator imunológico, principalmente o aloimune, foi o mais encontrado (93,9%). Mulheres com fator aloimune isolado obtiveram melhores resultados gestacionais (77.7% de partos) do que aquelas com a associação de outros fatores. A presença do fator autoimune aumentou a chance de aborto (RC 4.30 95% intervalo de confiança, IC 1.36 – 13.63). Não foi encontrada associação entre o número de abortos prévios ao tratamento e o resultado gestacional. Mulheres com 40 anos ou mais apresentaram a mais alta taxa de aborto espontâneo (OR 5.83 95% CI 1.12-30.40).

**CONCLUSÃO:**

Idade acima de 40 anos, a presença de fatores imunológicos e a associação de dois ou mais fatores conferiram o pior prognóstico gestacional às mulheres avaliadas.

## INTRODUCTION

The mechanisms involved in human reproduction are highly complex, especially the role played by immunological factors. Ten to 15% of clinically diagnosed pregnancies end in spontaneous abortion.^1^ Recurrent spontaneous abortion (RSA) is defined as three or more consecutive spontaneous pregnancy losses before the 20^th^ week of gestation, a situation that occurs in 1 to 2% of women of reproductive age.^2,3^

Chromosomal abnormalities are mentioned as responsible for 50 to 60% of spontaneous abortions in the first trimester,^4^ including numerical aberrations, translocations and mosaicism.^5^ Qualitative semen evaluation is currently suggested for investigations of the causes of RSA.^6^ Uterine anatomical defects and an incompetent cervix predispose women to abortion and premature delivery, accounting for 15 to 27% of RSA cases.^5,7^

Changes in hormonal patterns have also been implicated as causes of RSA. Low progesterone production renders endometrial maturation inadequate for nidation and egg development.^8,9^ Immunological alterations have been cited as responsible for more than 80% of RSA cases of unknown etiology.^10^ The discovery of an association between antiphospholipid antibodies and RSA was a major step towards preventing the occurrence of this complication.^11^

Similarly, it has been estimated that the alloimmune factor is present in 40% to 60% of couples with RSA of unknown etiology.^12^ The initial event is an abnormality that prevents the mother from developing the immunological response that is essential in relation to a genetically foreign conceptus.^13^ There seem to be decreased numbers of suppressor cells in the decidua and increased activity of natural killer cells (NK), which attack the implanted conceptus and thus increase the incidence of early abortions.^14^ The therapy proposed was to use the partner's or a donor's lymphocytes to suppress NK cell activity, making it possible for the conceptus to develop.^15^

Other factors, such as age and number of previous abortions are suspected causes of RSA. However, immunological factors appear to occur most frequently in women with RSA of unknown etiology.^16^

Despite the therapy available, it is still found today that some women who are regularly treated continue to experience recurrent abortions. Other complicating factors may exist in some groups, and it may also be that a combination of multiple causes contributes towards unsuccessful outcomes.

## OBJECTIVE

This study therefore sought to identify and treat these single or combined factors, and to evaluate pregnancy outcomes.

## PATIENTS AND METHODS

The gestational success rate described for women suffering from RSA who are treated for alloimmune causes ranges from 62 to 78%.^16–18^ If autoimmune causes are simultaneously treated, the expected gestational success rate is around 80%.^16–18^ Using these proportions, a sample of 246 RSA women would be sufficient for this study, with a 95% confidence interval (CI) and 5% type I error. This case-control study included medical records from 246 women with RSA who were seen at the Gestational Loss Outpatient Clinic of Universidade Estadual de Campinas, from March 1994 to July 2003. Among the 246 women who were initially included in the study, 17 had no records of gestational outcomes and were excluded, and thus the final number of women included in the study was 229.

The inclusion criteria were: history of three or more consecutive spontaneous abortions and becoming pregnant after completing the evaluation and treatment. The exclusion criteria were: induced gestational loss or not completing the investigation protocol. After data analysis, two unmatched groups were defined according to the gestational results: group 1 (deliveries) and group 2 (spontaneous abortions). The regular medical staff at the Gestational Loss Outpatient Clinic saw all the women included.

The investigation and treatment protocol included evaluation of genetic, hormonal, infectious, anatomical and immunological factors, as detailed in the following paragraphs.

Genetic factors were evaluated by karyo-typing, carried out on the couple. Those with abnormal results were evaluated by a clinical geneticist.

Hormonal factors were investigated by measuring serum progesterone and/or performing endometrial biopsy three to five days before menstruation. For all women with all factors identified who were advised that they could attempt conception (i.e. those in whom no autoimmunity had been detected), the use of natural progesterone (100 mg/day beginning at the second phase of the menstrual cycle) was recommended. The dose was doubled when pregnancy was confirmed and was maintained until the 16^th^ week of pregnancy.

Assessment of thyroid function was performed by measuring serum thyroid-stimulating hormone (TSH) and free thyroxine (FT4) levels. If hypothyroidism or hyperthyroidism was diagnosed, thyroid replacement or antithyroid drug therapy was used, respectively.

Diabetes mellitus (DM) was investigated by measuring fasting plasma glucose levels and performing a glucose tolerance test. Treatment of DM was achieved by diet follow-up or use of insulin when necessary.

Maternal infections were evaluated by culturing for *Mycoplasma* and *gonococci*, and by immunofluorescence and/or immunoenzymatic studies (for *Chlamydia*, syphilis and toxoplasmosis). When infection was confirmed, it was treated using well-known antibiotic or antiparasitic drugs.

Uterine anatomical defects were evaluated by clinical and gynecological examination and/or ultrasound and/or hysterosalpingography. When deemed necessary, the investigation was supplemented by hysteroscopy and/or laparoscopy, and surgical corrections needed were performed when possible.

The autoimmune factor was investigated by ELISA (enzyme-linked immunosorbent assay), to screen for immunoglobulin-G (IgG) and/or IgM anticardiolipin antibodies (aCL). Positive kaolin clotting time (KCT) and/or Russell viper venom time greater than 1.2 (and confirmed by a test diluted to 50%) were utilized to detect lupus anticoagulant activity (LAC).

Immunofluorescence was used to investigate antinuclear and anti-DNA antibodies. Treatment was based on the use of acetylsalicylic acid (ASA) and low molecular weight heparin. Investigation of the alloimmune factor was performed by crossmatch using the microlymphocyte toxicity test. The allo-immune factor was considered present when the crossmatch test between the female serum and the male partner's lymphocytes was negative. In these cases, serum tests for hepatitis B and C and for human acquired immunodeficiency virus (HIV), syphilis and Chagas disease were performed on both partners before treating this factor. If a diagnosis was made, immunization therapy was prepared, utilizing a concentration of 80 x 10^6^/ml of the male partner's lymphocytes intradermally injected into the female, on two occasions separated by a four to six-week interval. Cross-matching was repeated, and women who had demonstrated a partial response received two booster immunizations. A new crossmatch was performed to evaluate the response. Women who showed no response to immunization by their partner's lymphocytes (crossmatch remaining negative), or who did not present a change in partial response after two booster shots, were administered two immunizations using lymphocytes from a non-related donor together with the partner's lymphocytes. After a positive crossmatch, patients were then advised that they could attempt pregnancy.

Analysis of the association between morphological criteria and final diagnosis was performed using estimated odds ratio (OR) values, with 95% CI. This analysis was also performed using logistic regression models, and the results were expressed as OR values, with 95% CI. Decision trees were constructed, based on significant OR values. Statistics were calculated using the SAS 8.2 package (SAS Institute, Cary, NC).

The Research Ethics Committee of the institution gave its prior approval for this study.

## RESULTS

Among the 229 women studied, 200 had a history of three abortions, 24 had four or five, and five had six or more previous abortions. After application of the protocol and in accordance with the pregnancy results, the women in the study were divided into two groups. Group 1 included 170 women (74.2%) who went into labor and group 2 included 59 women (25.8%) whose pregnancy ended in spontaneous abortion. In group 1, 148 women (87%) had a full-term pregnancy, 22 women (13%) had preterm birth and six women had fetal death. In group 2, 51 women (86.4%) had one abortion and 8 (13.6%) had two abortions after treatment. Most of the women studied (86%) were 25 to 39 years old; the mean age in group 1 was 31.6 ± 4.8 years (range: 19 to 42) and in group 2 it was 32 ± 4.9 years (range: 20 to 43).

Alloimmune factor was found in 89.8% of the women who aborted and 95.3% of those who delivered. The second most frequently encountered factor was uterine. This occurred in 18.6% of the women who aborted and 17.6% of those who delivered. Autoimmune factor was detected in 16.9% of the women who aborted and 7.6% of those who delivered. Hormonal factor was detected in 8.5% of the women who aborted and 7.1% of those who delivered. Genetic factor was found in 5% of the women who aborted and 0.6% of those who delivered.

A variety of combinations of etiological factors were detected. There were cases with genetic factors, with associations between several factors and with no identified factors (the latter were classified as “others”). Single alloimmune factor was seen in 137 women (59.8%) and it was associated with other factors in 78 women (34%). Women with single alloimmune factor had better pregnancy outcomes (delivery) than women with associations between alloimmune and other factors. These women and those in the “others” group presented higher risk of abortion, as can be seen in Table 1.

**Table 1.** Odds ratios and percentage distribution of women with recurrent spontaneous abortion according to groups of factors investigated and first pregnancy outcome following treatment

Groups	Abortion (n = 59)		Delivery (n = 170)		Raw OR (95% CI)	Age-adjusted OR (95% CI)
	n	%	n	%		
**Single alloimmune**	28	47.5	109	64.1	Reference	Reference
**Alloimmune and autoimmune**	7	11.9	9	5.3	3.03 (1.04-8.84)	4.30 (1.36-13.63)
**Alloimmune and hormonal**	3	5.1	8	4.7	1.46 (0.36-5.86)	1.41 (0.34-5.96)
**Alloimmune and uterine**	5	8.5	22	12.9	0.89 (0.31-2.54)	1.13 (0.37-3.44)
**Non-immunological**	2	3.4	1	0.6	7.78 (0.68-88.95)	9.02 (0.70-116.5)
**Others**	14	23.7	21	12.4	2.60 (1.17-5.74)	2.79 (1.21-6.45)

*OR = odds ratio; CI = confidence interval.*

When the women studied were divided into groups according to age, it was observed that those aged 40 or older had a higher chance of spontaneous abortion (OR: 5.83; 95% CI: 1.12-30.40), with a negative influence on pregnancy outcome.

In evaluating the number of abortions prior to treatment, group 1 had an average of 3.5 abortions (range: 3 to 10) and group 2 had an average of 3.9 (range: 3 to 10). After correcting for the factors identified during the investigation, it was found that pregnancy outcome was not influenced by the number of abortions prior to treatment, and this can be seen in Table 2.

**Table 2.** Odds ratios and percentage distribution of women according to number of abortions prior to treatment and first pregnancy outcome following treatment

Number of abortions	Abortion (n = 59)		Delivery (n = 170)		Raw OR (95% CI)	Age-adjusted OR (95% CI)
	**n**	**%**	**n**	**%**		
**3**	48	81.4	152	89.4	Reference	Reference
**4 to 5**	9	15.3	15	8.8	0.94 (0.46-1.94)	0.96 (0.45-2.03)
**> 6**	2	3.4	3	1.2	2.37 (0.83-6.79)	2.66 (0.86-8.19)

*OR = odds ratio; CI = confidence interval.*

To view significant results better in order to perform multivariate analysis, decision trees were constructed to identify the sequences of events for evaluating the possible causes of RSA that might indicate better or worse pregnancy outcomes. These decision trees adjusted by event sequences were based on these criteria and the outcomes were arranged in a hierarchical order of odds ratios.

**Figure 1:**
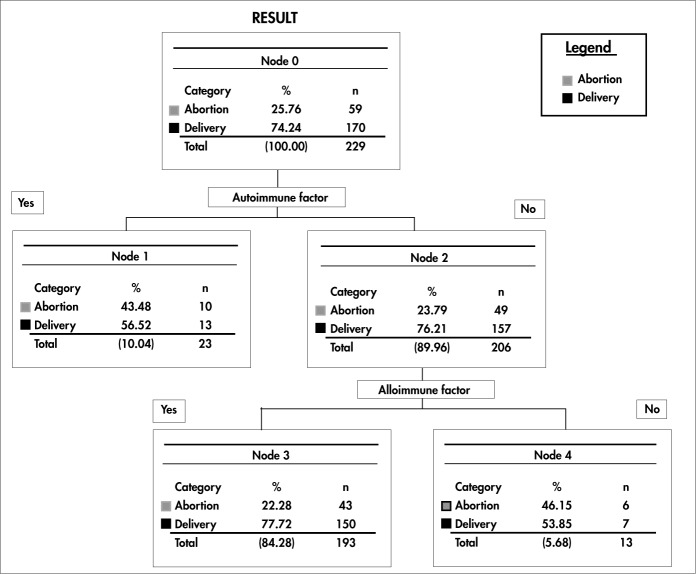
Decision tree regarding immunological, alloimmune and autoimmune factors, in which the left branches represent decisions made when the factor in question was found, and the right branches represent decisions made when this factor was absent.

Figure 1 shows the outcome after making “pruning” decisions that were supported by the desired clinical events. In agreement with the results from logistic regression, it was observed that the autoimmune factor variable was at the top of the decision levels. This criterion was followed by the alloimmune factor at the second level of decision. It was also noted that when the autoimmune factor was present, the percentage of deliveries decreased (from 74.2% to 56.5%), and thus the number of abortions increased (from 25.76% to 43.48%).

An increased percentage of deliveries (from 74.2% to 77.7%) and a reduced percentage of abortions (from 25.7% to 22.2%) were detected when the autoimmune factor was absent and the alloimmune factor was present (second-level decision). When both factors were absent, deliveries occurred in 53.8% and abortions in 46.1% of the women studied.

A new multivariate analysis was then performed to observe the groups or combinations of factors that were formed and related to RSA (alloimmune alone, alloimmune and autoimmune, alloimmune and hormonal, alloimmune and uterine, nonimmunological, and others).

**Figure 2:**
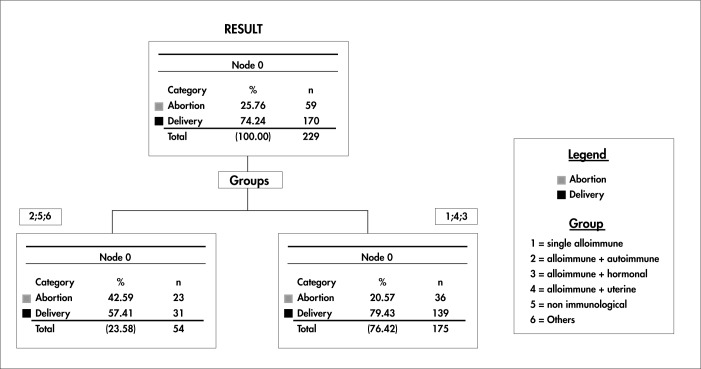
Decision tree regarding groups of factors investigated and grouped according to frequency.

The tree resulting from this new arrangement demonstrated the development of a decision level with groups of factors that, if present, worsened the outcome (more abortions), whereas others improved it (more deliveries). Under these conditions, as observed at the single level of this decision tree, women with an alloimmune factor alone or an allo-immune factor combined with hormonal or uterine factors had a better outcome (79.4% of deliveries and 20.5% of abortions). Despite this, women with associated alloimmune and autoimmune factors, those with nonimmunological factors and those with other factors (genetic factors, associations between several factors, and no identified factors) had a lower percentage of deliveries (57.4%) and a higher percentage of abortions (42.6%) than did all other possible combinations (Figure 2).

## DISCUSSION

In order to avoid a cohort effect, this study was based on a minimum investigation and treatment protocol for RSA. The pregnancy outcome in our institution, i.e. 74.2% deliveries and 25.8% abortions, was similar to that found in other clinics specializing in this type of care.^19^ The alloimmune factor was found in 93.9% of the cases and, when corrected, 77.7% of the women went into labor. Perhaps this frequency was higher than what was found by other authors because our institution is the only public health clinic in Brazil that investigates and treats women with RSA of alloimmune cause.

The immunological factor was the one most commonly found, and the data attested to its importance in the context of female reproductive health. The pregnancy outcome was better when the women presented a single alloimmune factor or an alloimmune factor combined with hormonal or anatomical factors. All the women who were advised that they could attempt conception received natural micronized progesterone. Thus, all the hormonal factors diagnosed were adequately treated, which may have contributed towards the better outcomes seen in the group with alloimmune combined with hormonal factors.

Likewise, anatomical factors were evaluated. The influence of these factors on the origin of RSA is still a matter of discussion in the literature.^5,7^ Nevertheless, when synechiae, uterine septa, polyps, myomas or cervical incompetence were diagnosed, treatment was given. It is possible that these corrections may have contributed towards better pregnancy outcomes among the women of the present study, as also reported by some other authors.^20,21^

On the other hand, worse outcomes were obtained among the women who had combined alloimmune and autoimmune factors, non-immunological factors and other factors (genetic factors, several associations and no identified factor).

Recent studies have confirmed that there is an association between antiphospholipid antibodies and RSA.^22^ Furthermore, they have reported that the combined use of aspirin and heparin has proven to be very efficient, and may reduce the chance of another abortion in 54% of the cases.^14–16^ The pregnancy outcomes related to the autoimmune factor found in the present study were quite similar to those presented in the literature, i.e. slightly more than 50% of the women went into labor.^23^

In agreement with other studies, we believe that the alloimmune factor is one of the main factors responsible for the RSA cases that are described as “of unknown etiology”.^19,23^ It is clear that when other factors besides the immunological ones are involved, new treatments need to be added and consequently the risks will be higher. The categories of combined alloimmune and autoimmune factors, non-immunological factors and other factors did not produce good gestational results. The “others” group was complex, since it involved women with no identified factors, several associations of factors, or genetic factors. The women in this group did not undergo specific therapeutic procedures and presented increased risk of abortion (OR: 2.79; 95% CI: 1.21-6.45). This complexity certainly contributed towards the worse outcomes in this group, since specific treatment could not be instituted and genetic therapy is unavailable in our country.

## CONCLUSION

Age over 40, immunological factors (particularly autoimmune factors), and two or more concomitant etiological factors were associated with poor gestational outcomes among the women studied.
